# Supplementary material for: Boosting the Concordance Index for Survival Data – A Unified Framework To Derive and Evaluate Biomarker Combinations
Source: PLoS One. 2014 Jan 6;9(1):e84483. doi: 10.1371/journal.pone.0084483 (PMC3882229; doi:10.1371/journal.pone.0084483)
Supplement: Text S1 — This document provides technical details on boosting the concordance index. It contains the implementation of the new Cindex() family and a short description of the necessary R Code to apply the algorithm in practice. (PDF) [file pone.0084483.s001.pdf]

Supporting information for
"Boosting the concordance index for survival data – a unified framework to derive and evaluate biomarker combinations"

Andreas Mayr[1], Matthias Schmid[2]

[1]Department of Medical Informatics, Biometry and Epidemiology,
Friedrich-Alexander-Universität Erlangen-Nürnberg, Erlangen, Germany
[2]Department of Statistics, Ludwig-Maximilians-Universität München, Munich, Germany

Gradient boosting of the C-index

The concept of boosting was first introduced in the field of machine learning [1, 2]. The basic idea is to boost the accuracy of a relatively weak performing classificator (termed "base-learner") to a more accurate prediction via iteratively applying the base-learner and aggregating its solutions. Generally, the concept of boosting leads to a drastically improved prediction accuracy compared to a single solution of the base-learner [3]. This basic concept was later adapted to fit statistical regression models in a forward stagewise fashion [4, 5]. One of the main advantages of this approach is the interpretability of the final solution, which is basically the same as in any other statistical model [6]. This can not be achieved with competing machine learning algorithms as Support Vector Machines [7] or Random Forests [8]. Specifically, the boosting approach can be used to develop prediction rules for survival outcomes [9–11]. Although there exist also likelihood-based approaches for boosting [12], we will her focus on gradient-based boosting [6] as it is the better fitting approach for boosting the distribution-free C-index.

The most flexible implementation of gradient boosting is the `mboost` [13] add-on package for the Open Source programming environment `R` [14]. The `mboost` package contains a large variety of different pre-implemented base-learners and loss functions that can be combined by the user via different fitting functions. For a tutorial on the how to apply the package for practical data analysis, see [15].

To apply gradient boosting to optimize linear biomarker combinations w.r.t. the C-index in the version of Uno et al. [16], it is necessary to specify the newly developed `Cindex()` family inside the `glmboost()` function.

The `Cindex` family object includes the sigmoid function $K(u) = 1/(1 + \exp(-u/\sigma))$ as approximation of the indicator functions in the estimated C-index. The sigmoid function is evaluated inside the `R` functions `approxGrad()` and `approxLoss()`, which are part of the `Cindex` object. The weights

$$w_{ik} \ := \ \frac{\Delta_i \, (\hat{G}_n^L(\tilde{T}_i))^{-2} \, \mathrm{I}\left(\tilde{T}_i < \tilde{T}_k\right)}{\sum_{i,k} \Delta_i \, (\hat{G}_n^L(\tilde{T}_i))^{-2} \, \mathrm{I}\left(\tilde{T}_i < \tilde{T}_k\right)} \tag{1}$$

are computed via the internal function `compute_weights()` for both the empirical risk

$$-\widehat{C}_{\mathrm{smooth}}(T, \eta) \ = \ -\sum_{i,k} w_{ik} \cdot \frac{1}{1 \ + \ \exp\left(\frac{\hat{\eta}_k - \hat{\eta}_i}{\sigma}\right)} \tag{2}$$

(implemented in the `risk()` function) as well as for the negative gradient

$$-\frac{\partial \, \widehat{C}_{\mathrm{smooth}}(T, \eta)}{\partial \, \eta_i} \ = - \sum_{k} w_{ik} \, \frac{-\exp\left(\frac{\hat{\eta}_k - \hat{\eta}_i}{\sigma}\right)}{\sigma \, \left(1 \ + \ \exp\left(\frac{\hat{\eta}_k - \hat{\eta}_i}{\sigma}\right)\right)} \tag{3}$$

(implemented in the `ngradient()` function).
Those different functions that define the optimization problem are finally plugged into the `mboost` specific `Family()` function to build a new `boost_family`. Details on how to implement user-specific families in `mboost` are presented in the Appendix of [15]. The complete `Cindex` object is then given as follows:

```
Cindex <- function (sigma = 0.1) {

      approxGrad <- function(x) {                     ## sigmoid function for gradient
        exp(x/sigma) / (sigma * (1 + exp(x/sigma))^2)
      }
      approxLoss <- function(x) {                     ## sigmoid function for loss
        1 / (1 + exp(x / sigma))
      }

      compute_weights <- function(y, w = 1){          ## compute weights
        ipcw_wow <- IPCweights(y[w != 0,])
        ipcw <- numeric(nrow(y))
        ipcw[w!=0] <- ipcw_wow
        survtime <- y[,1]
        n <- nrow(y)
        wweights <- matrix( (ipcw)^2, nrow = n, ncol = n)
        weightsj <- matrix(survtime, nrow = n, ncol = n)
        weightsk <- matrix(survtime, nrow = n, ncol = n, byrow = TRUE)
        weightsI <- (weightsj < weightsk) + 0
        wweights <- wweights * weightsI
        Wmat <- w %o% w
        wweights <- wweights * Wmat
        wweights <- wweights / sum(wweights)
        rm(weightsI); rm(weightsk); rm(weightsj)
      return(wweights)
      }

      ngradient = function(y, f, w = 1) {              ## negative gradient
        if (!all(w %in% c(0,1)))
          stop(sQuote("weights"), " must be either 0 or 1 for family ",
               sQuote("UnoC"))
        survtime <- y[,1]
        event <- y[,2]
        if (length(w) == 1) w <- rep(1, length(event))
        if (length(f) == 1) {
          f <- rep(f, length(survtime))
        }
        n <- length(survtime)
        etaj <- matrix(f, nrow = n, ncol = n, byrow = TRUE)
        etak <- matrix(f, nrow = n, ncol = n)
        etaMat <- etak - etaj
        rm(etaj); rm(etak);
        weights_out <- compute_weights(y, w)
        M1 <- approxGrad(etaMat) * weights_out
        ng <- colSums(M1) - rowSums(M1)
```

```
    return(ng)
  }

  risk = function(y, f, w = 1) {                   ## empirical risk
    survtime <- y[,1]
    event <- y[,2]
    if (length(f) == 1) {
      f <- rep(f, length(y))
    }
    n <- length(survtime)

    etaj <- matrix(f, nrow = n, ncol = n, byrow = TRUE)
    etak <- matrix(f, nrow = n, ncol = n)
    etaMat <- (etak - etaj)
    rm(etaj); rm(etak);
    weights_out <- compute_weights(y, w)
    M1 <- approxLoss(etaMat) * weights_out
    return(- sum(M1))
  }

    Family(                                         ## build the family object
      ngradient = ngradient,
      risk = risk,
      weights = "zeroone",
      offset = function(y, w = 1) {0},
      check_y = function(y) {
        if (!inherits(y, "Surv"))
          stop("response is not an object of class ", sQuote("Surv"),
               " but ", sQuote("family = UnoC()"))
        y},
      rclass = function(f){},
      name = paste("Concordance Probability by Uno")
      )
}
```

Application

We will briefly demonstrate how to apply the `Cindex` family in practice to derive the optimal combination of pre-selected biomarkers. We will use the van de Vijver et al. [17] data set of 144 lymph node positive breast cancer patients that was also considered in the main article. The data set is publicly available as part of the `R` add-on package `penalized` [18]. The 70-gene signature for metastasis-free survival after surgery was originally developed by van't Veer et al. [19].

We first split the data set in 100 training observations and 44 test observations. To ensure better readability of the code, we do not carry out stratified subsampling but just use the first 100 patients as training sample. Model fitting is carried out by the `glmboost()` function of the `mboost` package. As linear models are the default base-learners for `glmboost()`, no additional base-learner has to be specified.

As appropriate `family` object we specify the `Cindex` family described above.

For evaluating the discriminatory power of the resulting prediction on test data, we use the `UnoC()` function of the `survAUC` package [20]. It implements the unbiased estimator $\widehat{C}_{\text{Uno}}$, as proposed by Uno et al. [16].

```
## load add-on packages
library(penalized) ## for the data set
library(mboost)    ## for boosting
library(survAUC)   ## for evaluation

data(nki70)        ## loading the data

source("Cindex.R") ##  loading the family defined above

## split the data set in training and test sample (simplified):
dtrain <- nki70[1:100,]
dtest  <- nki70[101:144,]

## fit a model via the glmboost() function
## formula : defines the candidate model; the response is the survival
##           object Surv(time, event); via '~ ." all remaining variables
##           in the data set serve as possible predictors
## family  : defines the optimization problem (in this case the C-index)
##           sigma is the smoothing parameter of the sigmoid function that
##           approximates the indicator functions. The default value is 0.1.
## control : defines other boosting-specific tuning parameters like the
##           stopping iteration mstop or the step-length nu; trace = TRUE is
##           only for convenience (shows the trace of the empirical risk).
## data    : defines the data set -> training sample

mod1 <- glmboost(Surv(time,  event) ~ .,  family = Cindex(sigma = 0.1),
                 control = boost_control(mstop = 500, trace = TRUE, nu = 0.1),
                 data = dtrain)

## The stopping iteration can be changes via simple indexing:
mod1 <- mod1[50000] ## Long runtime: 50000 iterations
                    ## takes at least a couple of minutes on a standard machine

## Now take a look at the resulting combination
coef(mod1)

## Prediction on test data
preds <-  predict(mod1, newdata = dtest)

## Evaluate the discriminatory power
UnoC(Surv(dtrain$time, dtrain$event),  Surv(dtest$time, dtest$event), lpnew = -preds)
```

References

1. Schapire RE (1989) The Strength of Weak Learnability. CICS (Series). Massachusetts Institute of Technology. Laboratory for Computer Science Center for Intelligent Control Systems.

2. Freund Y (1990) Boosting a weak learning algorithm by majority. In: Fulk MA, Case J, editors, Proceedings of the Third Annual Workshop on Computational Learning Theory, COLT 1990, University of Rochester, Rochester, NY, USA, August 6-8, 1990. pp. 202–216.

3. Ridgeway G (1999) The state of boosting. Computing Science and Statistics 31: 172–181.

4. Friedman JH, Hastie T, Tibshirani R (2000) Additive logistic regression: A statistical view of boosting (with discussion). The Annals of Statistics 28: 337–407.

5. Buehlmann P (2006) Boosting for high-dimensional linear models. The Annals of Statistics : 559–583.

6. Bühlmann P, Hothorn T (2007) Boosting algorithms: Regularization, prediction and model fitting (with discussion). Statistical Science 22: 477–522.

7. Vapnik V (1996) The Nature of Statistical Learning Theory (Information Science and Statistics). Springer.

8. Breiman L (2001) Random forests. Machine Learning 45: 5–32.

9. Hothorn T, Bühlmann P, Dudoit S, Molinaro A, Van Der Laan M (2006) Survival ensembles. Biostatistics 7: 355–373.

10. Schmid M, Hothorn T (2008) Flexible boosting of accelerated failure time models. BMC Bioinformatics 9: 269.

11. Binder H, Allignol A, Schumacher M, Beyersmann J (2009) Boosting for high-dimensional time-to-event data with competing risks. Bioinformatics 25: 890–896.

12. Tutz G, Binder H (2006) Generalized additive modeling with implicit variable selection by likelihood-based boosting. Biometrics 62: 961–971.

13. Hothorn T, Bühlmann P, Kneib T, Schmid M, Hofner B (2013) mboost: Model-Based Boosting. URL `http://CRAN.R-project.org/package=mboost`. R package version 2.2-3.

14. R Core Team (2013) R: A Language and Environment for Statistical Computing. R Foundation for Statistical Computing, Vienna, Austria. URL `http://www.R-project.org/`. ISBN 3-900051-07-0.

15. Hofner B, Mayr A, Robinzonov N, Schmid M (2012) Model-based boosting in R: a hands-on tutorial using the R package mboost. Computational Statistics : 1–33.

16. Uno H, Cai T, Pencina MJ, D'Agostino RB, Wei LJ (2011) On the C-statistics for evaluating overall adequacy of risk prediction procedures with censored survival data. Statistics in Medicine 30: 1105–1117.

17. van de Vijver MJ, He YD, van't Veer LJ, Dai H, Hart AAM, et al. (2002) A gene-expression signature as a predictor of survival in breast cancer. New England Journal of Medicine 347: 1999–2009.

18. Goeman J (2012) penalized: L1 (Lasso) and L2 (Ridge) Penalized Estimation in GLMs and in the Cox Model. R package version 0.9-42.

19. van't Veer LJ, Dai HY, van de Vijver MJ, He YDD, Hart AAM, et al. (2002) Gene expression profiling predicts clinical outcome of breast cancer. Nature 415: 530–536.

20. Potapov S, Adler W, Schmid M (2012) survAUC: Estimators of Prediction Accuracy for Time-to-Event Data. R package version 1.0-5. http://cran.r-project.org/web/packages/survAUC/index.html.
